# Prognostic Value of Intraoperative Blood Transfusion in Patients with Adenocarcinoma of the Esophagogastric Junction

**DOI:** 10.3390/medicina58040474

**Published:** 2022-03-25

**Authors:** Kei Nakajima, Masanori Tokunaga, Keisuke Okuno, Katsumasa Saito, Naoto Fujiwara, Yuya Sato, Akihiro Hoshino, Takatoshi Matsuyama, Yusuke Kinugasa

**Affiliations:** Department of Gastrointestinal Surgery, Tokyo Medical and Dental University, 1-5-45, Yushima, Bunkyo-ku, Tokyo 113-8510, Japan; maxime16161616@gmail.com (K.N.); okuno.srg1@tmd.ac.jp (K.O.); katsumit14@gmail.com (K.S.); fujisrg1@tmd.ac.jp (N.F.); yusatoh.srg1@tmd.ac.jp (Y.S.); hosino.srg1@tmd.ac.jp (A.H.); matsuyama.srg1@tmd.ac.jp (T.M.); kinugasa.srg1@tmd.ac.jp (Y.K.)

**Keywords:** intraoperative blood transfusion, esophagogastric junction adenocarcinoma, survival outcomes, gastric cancer, esophageal cancer

## Abstract

*Background and objectives*: Adenocarcinoma of the esophagogastric junction (AEG) has a complicated surgical anatomy, due to which it sometimes induces excessive intraoperative blood loss that necessitates intraoperative blood transfusion (BTF). However, few reports have focused on the impact of BTF on the survival outcomes of patients with AEG. We aimed to evaluate the impact of BTF on AEG prognosis. *Materials andMethods*: We included 63 patients who underwent surgical resection for AEG at our hospital between January 2010 and September 2020. Clinicopathological characteristics and survival outcomes were compared between patients with (*n* = 12) and without (*n* = 51) BTF. Multivariate analysis was performed to identify the independent prognostic factors for overall survival. *Results*: None of the patients who underwent minimally invasive surgery received BTF. Patients who received BTF had a significantly worse 5-year survival rate than those who did not (67.8% vs. 28.3%, *p* = 0.001). BTF was an independent risk factor for overall survival (hazard ratio: 3.90, 95% confidence interval 1.30–11.7), even after patients who underwent minimally invasive surgery were excluded. *Conclusions*: BTF adversely affected the survival outcomes of patients with AEG who underwent curative surgery. To avoid BTF, surgeons should strive to minimize intraoperative bleeding.

## 1. Introduction

Gastric cancer is one of the most common malignancies, and several prognostic factors have been proposed. The adverse effects of intraoperative blood loss (IBL) and intraoperative blood transfusion (BTF) on survival outcomes following curative gastrectomy have been reported, but some of them failed to prove the negative impact of IBL and BTF on survival outcomes [1,2,3,4,5,6].

The incidence of adenocarcinoma of the esophagogastric junction (AEG) has been increasing in East Asia [7,8,9]. The complicated surgical anatomy around the esophagogastric junction makes surgery for AEG difficult. Compared to surgery for gastric cancers without esophageal infiltration, surgery for AEG has a longer duration and a higher incidence of postoperative complications. Additionally, excessive IBL that necessitates BTF may occur during surgery for AEG. However, few studies have focused on the effects of IBL and BTF in patients with AEG, and information regarding their impact on survival outcomes is limited [10,11,12,13,14,15].

Therefore, the objective of this study was to clarify the impact of IBL and BTF on the survival outcomes of patients with AEG undergoing curative surgery.

## 2. Material and Methods

This study included 81 consecutive patients with AEG who had undergone surgical resection at the Tokyo Medical and Dental University between January 2010 and September 2020. Patients who had undergone non-curative resection (eight patients), those who had undergone preoperative endoscopic submucosal dissection (nine patients), and those who had remnant stomach cancer (one patient) were excluded. The remaining 63 patients were included in the final analysis. Data regarding the patients’ characteristics, surgical and pathological findings, and clinical course were collected from our prospectively maintained database, and we referred to individual patient electronic medical records when necessary.

Pathological tumor depth, nodal status, and surgical curability were assessed based on the International Union Against Cancer TNM Classification of Malignant Tumors, eighth edition. The tumor epicenter was assessed through pathological examination, and the Siewert classification was used for AEG categorization.

This retrospective study was conducted in accordance with the Declaration of Helsinki, and the study protocol was approved by the institutional review board of the Tokyo Medical and Dental University (No. M2020-279; approved date, December 16, 2020). Written informed consent was waived in this retrospective study.

### 2.1. Comparison between Patients with and without BTF

Clinicopathological characteristics and survival outcomes were compared between patients who required BTF (BTF group, *n* = 12) and those who did not (non-BTF group, *n* = 51).

### 2.2. Statistical Analysis

Continuous data are presented as medians (ranges), and they were compared using the Mann–Whitney U test. Categorical data were compared using Fisher’s exact test. Overall survival (OS) was calculated as the time from the date of surgery to the date of the last observation or death. Survival curves were derived from Kaplan–Meier estimates, and the curves were compared using the log-rank test. As all patients who had undergone minimally invasive surgery were in the non-BTF group, survival curves were also compared after excluding the patients who had undergone minimally invasive surgery. Prognostic factors were identified using the multivariable Cox proportional hazards model. Covariates with *p* < 0.1 in the univariate analysis were used as covariates in the subsequent multivariable analysis. Statistical significance was set at *p* < 0.05. All statistical analyses were performed using R statistics version 4.0.3 (R development core team, Vienna, Austria) [16].

## 3. Results

### 3.1. Clinicopathological Characteristics

Leukocyte-reduced red blood cell concentrates were transfused in all 12 patients of the BTF group, with a median transfusion amount of 560 mL (280–1120 mL). Fresh frozen leukocyte-reduced plasma was transfused in four patients, with a median transfusion amount of 480 mL (240–960 mL). The comparisons of the patient characteristics, surgical and pathological findings, and short-term postoperative results between the BTF and non-BTF groups are shown in Table 1, Table 2, Table 3 and Table 4. There were no between-group differences in sex or age. The preoperative hemoglobin level was significantly lower in the BTF group than in the non-BTF group (Table 1). The transthoracic approach was more frequently used in the BTF group than in the non-BTF group. All BTF patients underwent open surgery. On the other hand, 29 patients in the non-BTF group underwent minimally invasive surgery, and 22 of them received open surgery (Table 2). The tumor size was larger and the length of esophageal invasion was longer in the BTF group than in the non-BTF group (Table 3). The incidence of Clavien–Dindo grade IIIA postoperative complications was 58.3% and 17.6% in the BTF and non-BTF groups, respectively (*p* = 0.117, Table 4). More patients received adjuvant chemotherapy in the BTF group (60%) than in the non-BTF group (42%), although this difference was not statistically significant (*p* = 0.332, Table 1).

### 3.2. Survival Data and Prognostic Factors

**Figure 1:**
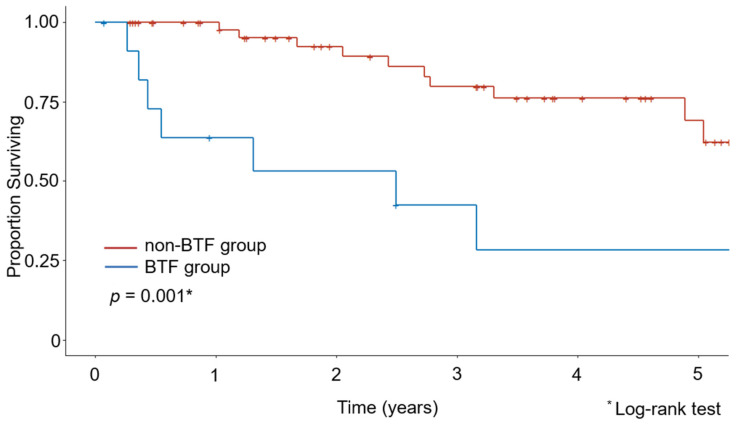
Kaplan–Meier estimates of overall survival in the intraoperative blood transfusion (BTF) and non-BTF groups.

**Figure 2:**
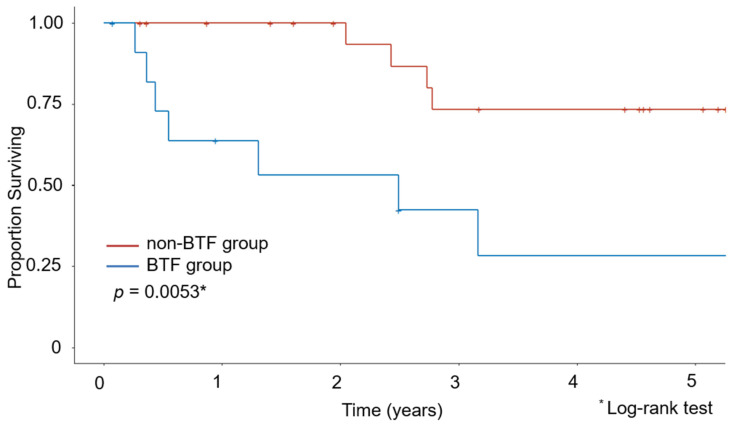
Kaplan–Meier estimates of overall survival after excluding patients who underwent minimally invasive surgery.

The Kaplan–Meier curves for OS are shown in Figure 1 and Figure 2. The median observation period for survivors was 29.6 months. The non-BTF group had a better 5-year survival rate than the BTF group (67.8% vs. 28.3%, *p* = 0.001). Even after excluding the patients who had undergone minimally invasive surgery, the non-BTF group had a better 5-year survival rate than the BTF group (73.3% vs. 28.3%, *p* = 0.005).

### 3.3. Univariate and Multivariate Analysis of Prognostic Factors

Table 5 shows the results of the Cox proportional hazards model for OS. In the univariate analysis, pathologic stage, BTF, and vascular invasion were found to be potential prognostic factors. Multivariate analysis using these three covariates identified only BTF as an independent prognostic factor (hazard ratio: 3.90, 95% confidence interval 1.30–11.7). After excluding the patients who had undergone minimally invasive surgery, duration of surgery and BTF were identified as independent prognostic factors in univariate analysis (Table 6). The subsequent multivariable analysis using these two covariates identified only BTF as an independent prognostic factor (hazard ratio, 5.04, 95% confidence interval 1.42–18.0).

## 4. Discussion

Perioperative blood transfusion has been reported to be associated with poor postoperative survival outcomes in patients with gastric cancer and those with colorectal cancer [2,3,4,17]. We believe that this is the first report that only included patients with AEG and investigated the relationship between BTF and survival outcomes [10,11,12,13,14,15]. Our findings demonstrate the negative impact of BTF on the survival outcomes of patients with AEG.

BTF-induced anti-tumor immunosuppression may explain the adverse effect of BTF on survival outcomes. In 1981, Gantt reported that perioperative allogeneic blood transfusion-induced immunosuppression might promote tumor growth [18]. Since then, many studies have demonstrated that BTF can cause a wide range of cytokine-mediated immune responses and suppress cellular and humoral immunity [19,20,21,22]. BTF-induced immunomodulatory effects include a reduction in the levels of interferon-gamma [19] and T-lymphocyte subsets (CD3+, CD4+, and CD4+/CD8+) [19,21,22], and suppression of interleukin-2 production [20], which may worsen survival outcomes.

There were some between-group differences in clinicopathological characteristics. The BTF group included patients with large and advanced-stage tumors; further, the duration of surgery was longer and the incidence of postoperative complications was higher in the BTF group than in the non-BTF group. These factors can affect survival outcomes; therefore, to identify independent prognostic factors, we conducted multivariate analysis using the possible prognostic factors identified by univariate analysis. However, our study sample was relatively small, and another study with a larger sample should be conducted to confirm our results.

In this study, none of the patients who had undergone minimally invasive surgery, including robotic or laparoscopic surgery, required BTF. Although minimally invasive surgeries have a longer duration of surgery than open surgeries, they are associated with less intraoperative blood loss, resulting in a decreased requirement for perioperative blood transfusion [23]. To eliminate the effect of the surgical approach, we compared the survival curves and identified independent prognostic factors after excluding the patients who had undergone minimally invasive surgery; we found that patients with BTF had poor survival outcomes, and BTF was identified as an independent prognostic factor.

This study has some limitations. First, it was a single-center, retrospective study. The sample size was small, and due to insufficient power, we could not assess the impact of BTF according to Siewert type. Second, some patients were followed for less than five years. To validate the results of this study, a well-designed prospective, multicenter study is warranted. Significant differences in clinicopathologic characteristics exist between the non-BTF and BTF groups. To eliminate potential bias between the groups, we conducted subgroup and multivariate analyses. However, the differences could not be completely adjusted for, and therefore a well-designed multicenter study with a larger number of patients is warranted.

## 5. Conclusions

BTF was significantly associated with poor OS in patients with AEG undergoing curative surgery. Surgeons should strive to minimize IBL to avoid BTF, which may adversely affect survival outcomes.

## Figures and Tables

**Table 1:** Patient characteristics.

	Non-BTF Group	BTF Group	*p* Value
Age			
Median	67	67	0.720 **
Range	46–89	29–82	
Sex			
Female	40	10	0.684 *
Male	11	2	
Preoperative BMI			
Median	23.78	21.56	0.096 **
Range	15.29–41.17	16.70–28.45	
CONUT score			
≦2	42	9	0.684 *
≧3	9	3	
Preoperative Hb			
Median	13.2	12.15	0.007 **
Range	9.90–17.20	8.40–14.20	
Neoadjuvant chemotherapy			
No	48	12	1.000 *
Yes	3	0	
Adjuvant chemotherapy			
No	20	7	0.332 *
Yes	31	5	

* Fisher’s exact test; ** Mann–Whitney U test; BTF: intraoperative blood transfusion; BMI: body mass index; CONUT score: Controlling Nutritional Status score; Hb: hemoglobin.

**Table 2:** Surgical findings.

	Non-BTF Group	BTF Group	*p* Value
Surgical approach			
Transhiatal	31	3	0.050 *
Transthoracic	20	9	
Surgical procedure			
Minimally invasive surgery	29	0	<0.001 *
Open surgery	22	12	
Duration of surgery			
Median	376	329	0.076 **
Range	177–689	225–444	
Intraoperative blood loss			
Median	256	924	<0.001 **
Range	0–1784	318–3355	

* Fisher’s exact test; ** Mann–Whitney U test; BTF: intraoperative blood transfusion.

**Table 3:** Pathological findings.

	Non-BTF Group	BTF Group	*p* Value
Histology			
Differentiated	26	7	0.756 *
Undifferentiated	24	5	
Siewert classification			
Type I	1	4	<0.001 *
Type II/III	50	8	
Esophageal invasion length			
<10 mm	20	0	0.012 *
≧10 mm	31	12	
Tumor size			
Median	53	95	<0.001 **
Range	13–140	53–145	
pStage (UICC)			
IB	9	0	0.296 *
IIB	4	1	
IIIA	2	0	
IIIB	21	4	
IVA	15	7	
pStage (UICC)			
≦IIIB	36	5	0.091 *
≧IVA	15	7	
Vascular invasion			
No	17	2	0.318 *
Yes	34	10	

* Fisher’s exact test; ** Mann–Whitney U test; BTF: intraoperative blood transfusion; pStage: pathologic stage; UICC: International Union Against Cancer.

**Table 4:** Short-term postoperative results.

	Non-BTF Group	BTF Group	*p* Value
Clavien–Dindo classification			
≦grade II	42	7	0.117 *
≧grade III	9	5	
Postoperative hospital stay			
≦12 days	28	1	0.004 *
>12 days	23	11	
Neoadjuvant chemotherapy			
No	48	12	1.000 *
Yes	3	0	
Adjuvant chemotherapy			
No	20	7	0.332 *
Yes	31	5	

* Fisher’s exact test; BTF: intraoperative blood transfusion.

**Table 5:** Cox proportional hazards model for overall survival.

	Univariate Analysis			Multivariate Analysis		
	HR	95% CI	*p* Value *	HR	95% CI	*p* Value *
Age (<67 years vs. ≧67 years)	1.63	0.600–4.40	0.339	-	-	-
Sex (female vs. male)	0.828	0.291–2.36	0.724	-	-	-
Preoperative BMI (≦23.5 vs. >23.5)	0.722	0.273–1.91	0.512	-	-	-
CONUT score (≦2 vs. ≧3)	1.74	0.553–5.47	0.343	-	-	-
Preoperative Hb (≧12 vs. <12)	1.29	0.480–3.51	0.608	-	-	-
Surgical approach (transhiatal vs. transthoracic)	0.847	0.326–2.20	0.733	-	-	-
Surgical procedure (minimally invasive vs. open)	1.37	0.502–3.72	0.542	-	-	-
Histology (differentiated vs. undifferentiated)	0.891	0.343–2.31	0.812	-	-	-
Siewert classification (type II/III vs. type I)	2.32	0.526–10.2	0.267	-	-	-
pStage (UICC) (≦IIIB vs. ≧IVA)	2.86	1.08–7.60	0.035	1.27	0.409–3.94	0.681
Duration of surgery (≦6 h vs. >6 h)	1.91	0.713–5.11	0.198	-	-	-
Intraoperative blood loss (≦340 mL vs. >340 mL)	1.53	0.581–4.04	0.389	-	-	-
Intraoperative transfusion (no vs. yes)	4.43	1.67–11.7	0.003	3.9	1.30–11.7	0.015
Clavien–Dindo classification (≦grade II vs. ≧grade III)	1.48	0.478–4.57	0.497	-	-	-
Postoperative hospital stay (>12 days vs. ≦12 days)	2.13	0.750–6.06	0.156	-	-	-
Vascular invasion (no vs. yes)	6.51	0.862–49.1	0.070	5.65	0.687–46.4	0.107
Esophageal invasion length (≦10 mm vs. >10 mm)	1.84	0.524–6.46	0.341	-	-	-
Adjuvant chemotherapy (no vs. yes)	0.734	0.258–2.09	0.563	-	-	-

* Cox proportional hazards model; HR: hazard ratio; CI: confidence interval; CONUT score: Controlling Nutritional Status score; Hb: hemoglobin; pStage: pathologic stage; UICC: International Union Against Cancer.

**Table 6:** Cox proportional hazards model for overall survival (excluding patients who had undergone minimally invasive surgery).

	Univariate Analysis			Multivariate Analysis		
	HR	95% CI	*p* Value *	HR	95% CI	*p* Value *
Age (<67 years vs. ≧67 years)	2.13	0.563–8.10	0.265	-	-	-
Sex (female vs. male)	1.18	0.254–5.52	0.830	-	-	-
Preoperative BMI (≦23.5 vs. >23.5)	0.694	0.211–2.28	0.548	-	-	-
CONUT score (≦2 vs. ≧3)	1.67	0.441–6.31	0.451	-	-	-
Preoperative Hb (≧12 vs. <12)	1.36	0.396–4.66	0.625	-	-	-
Surgical approach (transhiatal vs. transthoracic)	0.978	0.258–3.71	0.975	-	-	-
Histology (differentiated vs. undifferentiated)	0.839	0.245–2.87	0.779	-	-	-
Siewert classification (type II/III vs. type I)	1.99	0.425–9.29	0.382	-	-	-
pStage (UICC) (≦IIIB vs. ≧IVA)	1.88	0.570–6.21	0.300	-	-	-
Duration of surgery (≦6 h vs. >6 h)	3.04	0.911–10.2	0.070	3.12	0.882–11.1	0.078
Intraoperative blood loss (≦340 mL vs. >340 mL)	3.3	0.418–26.1	0.257	-	-	-
Intraoperative transfusion (no vs. yes)	4.93	1.43–17.0	0.012	5.04	1.42–18.0	0.013
Clavien–Dindo classification (≦grade II vs. ≧grade III)	1.99	0.524–7.52	0.312	-	-	-
Postoperative hospital stay (>12 days vs. ≦12 days)	4.69	0.599–36.7	0.141	-	-	-
Vascular invasion (no vs. yes)	3.88	0.494–30.4	0.197	-	-	-
Esophageal invasion length (≦10 mm vs. >10 mm)	1.82	0.231–14.3	0.570	-	-	-
Adjuvant chemotherapy (no vs. yes)	1.24	0.377–4.07	0.724	-	-	-

* Cox proportional hazards model; HR: hazard ratio; CI: confidence interval; CONUT score: Controlling Nutritional Status score; Hb: hemoglobin; pStage: pathologic stage; UICC: International Union Against Cancer.

## Data Availability

The data presented in this study are available on request from the corresponding author. The data are not publicly available due to ethical restrictions.
